# Association of cigarette and e-cigarette use with cannabis-related risk perceptions and intentions

**DOI:** 10.1186/s42238-025-00288-6

**Published:** 2025-05-31

**Authors:** Ronja Kleine, Barbara Isensee, Frauke Nees, Reiner Hanewinkel

**Affiliations:** 1 Institute for Therapy and Health Research, IFT-Nord, Kiel, Germany; 2 Institute of Medical Psychology and Medical Sociology, University Medical Center Schleswig-Holstein, University, Kiel, Germany

**Keywords:** Cannabis, Combustible cigarette, E-cigarette, Risk perception, Use intention, Adolescence

## Abstract

**Background:**

This study examines the associations of current use of combustible cigarettes or e-cigarettes with perceived risk of cannabis use and intention to use cannabis within the next year.

**Methods:**

Data from 8,521 German adolescents were collected between autumn 2021 and spring 2022. Former but not current users of cannabis or (e-)cigarettes were excluded from analyses. Mean age was 14.0 years, and 50.6% of the sample were female. Current cannabis use was reported by 5.8%, current use of combustible and/or e-cigarettes by 17.0%. We conducted linear regression models predicting 1) risk perception by (e-)cigarette use, and 2) intention to use cannabis by (e-)cigarette use and risk perception, among never cannabis users.

**Results:**

Use of cannabis and (e-)cigarettes were strongly associated. Perceiving cannabis as less harmful was associated with a higher intention to use cannabis. Among never cannabis users, dual users of combustible and e-cigarettes perceived cannabis as least harmful and had the highest intention to use cannabis, followed by combustible cigarette-only users, and then e-cigarette-only users. Never combustible nor e-cigarette users perceived cannabis as most harmful and had the lowest intention to use cannabis.

**Conclusion:**

Perception of cannabis as harmful might be a protective factor for intentions to use cannabis among German adolescents. Prevention programs should consider that adolescent combustible and e-cigarette users are a high-risk group for initiation of cannabis use.

**Supplementary Information:**

The online version contains supplementary material available at 10.1186/s42238-025-00288-6.

## Background

Cannabis plays an increasingly important role in Germany as it has been partially legalized in April 2024 and is commonly used with growing prevalences over the past decade (European Monitoring Centre for Drugs and Drug Addiction 2023; Orth and Merkel 2021; Rauschert et al. 2022; Seitz et al. 2019). This occurs especially among young people: In 2021, 7.6% of adolescents and 25.0% of young adults used cannabis, compared to 4.6% and 13.5% in 2011 (Orth and Merkel 2021). This is a cause of concern as previous studies have shown that early, frequent, and heavy cannabis users are at high risk of poor cognitive and psychiatric outcomes (e. g. mood disorders, psychotic symptoms, cannabis use disorders, neurocognitive impairments) as well as impaired physical health (e. g. cardiovascular and respiratory diseases) (Hoch et al. 2015; Karila et al. 2014; Levine et al. 2017).

### Cannabis and tobacco co-use

Cannabis use has been shown to be associated with the use of combustible cigarettes and e-cigarettes (Ramo et al. 2012). Strong et al. (2018) identified the lowest rates of current cannabis use among non-tobacco-users (3.7%), followed by 14.6% among e-cigarette-only, 16.2% among combustible cigarette-only and 19.3% among combustible cigarette and e-cigarette users. Among German adolescents and adults (Kotz et al. 2024), current cigarette users were 9 times and current e-cigarette users were 5 times more likely to currently use cannabis compared to never users of each product, and 82.8% of cannabis users smoked cannabis together with tobacco in e. g. joints.

Different theories about the causality of the association are reviewed by Lemyre, Poliakova, and Bélanger (Lemyre et al. 2019), amongst them the gateway theory which assumes that the use of combustible/e-cigarettes serves as a gateway to later use of cannabis. This theory is supported by different studies’ findings: Youths who used combustible/e-cigarettes reported higher odds of later cannabis use initiation in observational studies (Ramo et al. 2012; Chadi et al. 2019; Seidel et al. 2022; Sun et al. 2022). Nevertheless, there exist other theories, such as shared genetic or environmental predispositions, co-administration of cannabis and tobacco, as well as growing evidence for a reverse gateway theory (Lemyre et al. 2019; Agrawal et al. 2012).

Co-use of tobacco and cannabis is problematic as it is associated with several negative outcomes beyond the once associated with single use of cannabis or tobacco, e. g. higher risk of dependency symptoms (Ramo et al. 2012), mental health problems (Hindocha et al. 2021), physical health problems e. g. cardiovascular and respiratory problems (Agrawal et al. 2012), risk behaviors (Bingham and Shope 2004; McCaffrey et al. 2010; Parkes et al. 2007) and neurocognitive impairment (Fried et al. 2006; Jacobsen et al. 2007).

### Cannabis risk perception

While prevalence of cannabis use increased over the last decade there was a decrease in adolescents’ perception of cannabis as harmful (Levy et al. 2021). Perception of cannabis as harmful has consistently been found to be negatively correlated with cannabis use (Lemyre et al. 2019; Salloum et al. 2018). Two reciprocal theories about the association between risk perception and cannabis use are discussed (Grevenstein et al. 2015): decreases in risk perception lead to increases in use (motivational hypothesis), and positive cannabis use experiences lead to less perceived risk (risk reappraisal hypothesis). Salloum et al. (2018) found associations in both directions with stronger evidence for the motivational hypothesis. Among never cannabis users, perceiving cannabis as less harmful was associated with expressing a positive intention to use cannabis within the next year (Lopez-Quintero and Neumark 2010).

Only few studies examined the influence of tobacco use on cannabis risk perception. As reported by Berg et al. (2015), current tobacco use was associated with a less harmful perception of not only tobacco but also cannabis products.

### Study aims

Some countries like Uruguay, Canada and several US-American states have begun to legalize the sale, possession, and use of cannabis for recreational purposes (United Nations Office on Drugs and Crime (UNODC) 2022). In Germany, medical prescription of cannabis is allowed since 2017, and in April 2024 the German government passed a first partial legalization of recreational use of cannabis. Under certain conditions, recreational use of cannabis is no longer illegal for above 18-year-olds in Germany. One key objective is to improve the protection of young people through systematic education and prevention (Federal Government Germany 2023). For successful implementation of such measures an important precondition is in-depth knowledge about the risk factors for cannabis use to properly address them, amongst them perception of cannabis as harmless. Since literature shows high evidence for an association between cannabis use and the use of various tobacco products, it can be assumed that tobacco use plays a crucial role in the prevention of cannabis use (e. g. targeting this risk group, educate about the risks of co-use).

This study aims to examine associations between current tobacco use, cannabis risk perception and intention to use cannabis. Previous research has mainly been limited to the US and to adults. This study’s aim is to provide new information from a different country and for a much younger sample. Further, to our knowledge, there exist only few studies examining the relationship between tobacco use and cannabis risk perception.

We hypothesize that, among never cannabis users, 1) current combustible or e-cigarette users perceive a lower risk of cannabis use and 2) have a higher intention to use cannabis compared to never users, and 3) the intention to use cannabis is negatively linked with risk perception.

## Methods

### Data

A secondary analysis of data gathered from two trials evaluating the effect of two school-based cannabis prevention programs (“Quo Vadis”, “Methodenkoffer”) addressing students in grades 8 and 9 was conducted. In this work we focussed on the respective baseline-data that has been collected between autumn 2021 and spring 2022.

### Participants

Students were recruited in 9 of 16 German states. In total, 1,615 schools with students in grades 8 and 9 were invited to participate. The available data originates from 554 classes in 115 schools, resulting in *N* = 8,521 observations. For further information see Appendix – Fig. 1.

### Ethics

Both studies were approved by the ethics committee of the German Society of Psychology (Reference: HanewinkelReiner2020-02-03VA, HanewinkelReiner2021-05-25WV). Prior to implementation, approval was obtained from the relevant authorities in the participating states and the schools consented to participate. Individuals’ participation required written informed consent from the parent/guardian as well as the participating students.

### Measures

Baseline-data collection took place in class with online or paper pencil questionnaires. Assessment domains relevant to the current study included sociodemographic characteristics, patterns of cannabis, combustible cigarette and e-cigarette use, risk perception of cannabis use, and intention to use cannabis. Current alcohol use, hookah use and peers’ cannabis use were considered as control variables.

#### Demographics

Sociodemographic variables included age, gender (binary: male, female), student’s and parents’ migration history (yes/no), and subjective social status assessed with the MacArthur scale (Goodman et al. 2001) from 1 “the least money, least education, worst jobs, or no job” to 10 “the most money, most education, and best jobs”.

#### Cannabis use

Lifetime use of cannabis was assessed as follows: “How often did you use cannabis in your lifetime.” Participants that answered “never” were named “never cannabis users” (*n* = 7,405). Participants that answered “once”, “two to five times” or “more than five times” were asked about their current cannabis use: “How often do you use cannabis currently?”. Participants’ answer “not at all” was grouped as “former cannabis users” (*n* = 387), all other answers (“less frequently than once a month”, “at least once a month, not weekly”, “at least once a week, not daily”, “(nearly) daily”) were grouped as “current cannabis users” (*n* = 456). Number of missing data was *n* = 270 (3.2%).

#### Combustible cigarette and e-cigarette use

Lifetime use of combustible cigarettes was assessed as follows: “Have you ever smoked cigarette?” (“yes”, “no”), use of e-cigarettes respectively. Participants that never smoked combustible cigarettes nor e-cigarettes were named “never combustible nor e-cigarette user” (*n* = 5,663). Current use of combustible cigarettes was assessed with the question: “Have you smoked cigarette in the past 30 days?” (“yes”, “no”), use of e-cigarettes respectively. Past 30-day users of combustible cigarettes but no e-cigarettes were named “current combustible cigarette users” (*n* = 430), vice versa “current e-cigarette users” (*n* = 463). Participants that used combustible cigarettes and e-cigarettes in the past 30 days were named “current dual users” (*n* = 584). Lifetime users of combustible and/or e-cigarettes with no past-30-day-use of neither substance were called “former combustible/e-cigarette users” (*n* = 1,253). Number of missing data was *n* = 125 (1.5%).

#### Cannabis risk perception

Risk perception of cannabis use was assessed with five items: “How much do you think people risk harming themselves if they… try cannabis/use cannabis sometimes/use cannabis regularly/use cannabis daily/use cannabis when they have problems (e. g. conflicts)?” Likert scale response options included 0 “no risk at all”, 1 “slight risk”, 2 “moderate risk”, 3 “great risk”, 4 “very high risk”. An overall mean was calculated as indicator for cannabis risk perception, higher scores indicating greater perceived harm. Internal consistency reliability was good (Cronbach’s alpha = 0.87).

#### Cannabis use intention

Intention to use cannabis was assessed with two items differing slightly between both studies: “Do you think, you will use cannabis… next weekend/the next year?” (Quo Vadis), “Do you think, you will use cannabis… the next two weeks/the next half year?” (Methodenkoffer). Likert scale response options included 0 “certainly no”, 1 “probably no”, 2 “probably yes”, 3 “certainly yes”. An overall mean was calculated as indicator for cannabis use intention, higher scores indicating greater intention to use cannabis in the near future. Internal consistency reliability was acceptable for Quo-Vadis (Cronbach’s alpha = 0.78) and good for Methodenkoffer (Cronbach’s alpha = 0.87).

#### Control variables

Current use of alcohol was assessed with the question: “Have you drunk alcohol in the past 30 days?” (“yes”, “no”), current use of hookah respectively: “Have you smoked hookah in the past 30 days?” (“yes, “no”). Information about cannabis use within the peer-group was assessed with the question: “How many of your friends use cannabis?” (0 = “none”, 1 = “few”, 2 = “about half”, 3 = “many”, 4 = “(almost) all”).

### Data analysis

Statistical analysis was conducted with R 4.3.1 (R: a language and environment for statistical computing 2023). The groups former cannabis users and former (e-)cigarette users (*n* = 1,532) were excluded due to potentially unknown confounding variables, e. g. the reason for quitting. Observations with missing in at least one of the grouping variables were dropped as well (*n* = 275). Three observations were excluded due to inconsistent data in lifetime and past 30-day (e-)cigarette use. This resulted in a sample size of *N* = 6,711.

Joint distribution of (e-)cigarette use and cannabis use was examined. We calculated the adjusted odd ratios (aORs) of current cannabis use with the different current (e-)cigarette consumption groups compared to never combustible nor e-cigarette users.

Complete-case linear regressions were performed to examine 1) the association between the (e-)cigarette consumption groups (predictor) and cannabis use risk perception (criterium), and 2) the association between the (e-)cigarette consumption groups, risk perception (predictors) and intention to use cannabis (criterium). Regressions were calculated with never cannabis users only. Linear models were defined with main effects only. The categorical predictor (e-)cigarette use was dummy coded with never users being the reference group. Models were adjusted for source of the data to account for item differences between both studies. Standardized regression coefficients with 95% confidence intervals (95%-CI) are reported. Post-hoc estimated means and pairwise contrasts were calculated adjusting *p*-values with Tukey’s method.

Demographic characteristics, current alcohol use, current hookah use and peers’ cannabis use are reported descriptively but were not included in the regression models at first hand due to missing data concerning 7.7% of the observations (between 0.05% within current hookah use and 3.4% within migration history). Sensitivity analyses were done to compare the reported results with corresponding complete-case models that are adjusted for control variables.

## Results

### Sample characteristics

Among the 8,521 observations, the analytic sample included those who were identified as never cannabis users (*n* = 6,320) or current cannabis users (*n* = 391) and being either never combustible nor e-cigarette users (*n* = 5,573), current e-cigarette users (*n* = 387), current combustible cigarette users (*n* = 317) or current dual users (*n* = 434). Weighted demographic characteristics (gender, age, migration history, social status), current alcohol and hookah use, and peers’ cannabis use within the different cannabis and (e-)cigarette use groups are presented in Table 1.

**Table 1:** Descriptives of cannabis and combustible cigarette/e-cigarette users

Variable	Never cannabis user												Current cannabis user											
	Never combustible nor e-cigarette user			Current e-cigarette user			Current combustible cigarette user			Current dual user			Never combustible nor e-cigarette user			Current e-cigarette user			Current combustible cigarette user			Current dual user		
	n	% col	% row	n	% col	% row	n	% col	% row	n	% col	% row	n	% col	% row	n	% col	% row	n	% col	% row	n	% col	% row
Gender																								
Female	2,807	51.3	82.7	160	46.6	4.7	129	62.3	3.8	112	58.6	3.3	6	42.9	0.2	10	29.4	0.3	55	59.1	1.9	115	50.9	3.4
Male	2,662	48.7	83.6	183	53.3	5.7	78	37.7	2.5	79	41.3	2.5	8	57.1	0.3	24	70.6	0.8	38	40.9	1.2	111	49.1	3.5
Age group																								
< 13	23	0.4	88.5	1	0.2	3.8	1	0.4	3.8	-	-	-	-	-	-	-	-	-	-	-	-	1	0.4	3.8
13–14	4,305	77.6	86.7	194	55.6	3.9	151	70.2	3.0	116	58.9	2.3	7	50.0	0.1	20	52.6	0.4	55	53.9	1.1	116	49.4	2.3
15–16	1,189	21.4	71.7	148	42.4	8.9	61	28.4	3.7	79	40.1	4.8	7	50.0	0.4	15	39.5	0.9	45	44.1	2.7	115	48.9	6.9
> 16	29	0.5	61.7	6	1.7	12.8	2	0.9	4.3	2	1.0	4.3	-	-	-	3	7.9	6.4	2	2.0	4.3	3	1.3	6.4
Migration history																								
No	3,773	70.4	83.1	201	59.5	4.4	176	84.2	3.9	130	69.1	2.9	10	71.4	0.2	21	61.8	0.5	68	68.0	1.5	161	71.9	3.5
Yes	1,589	29.6	82.4	137	40.5	7.1	33	15.8	1.7	58	30.9	3.0	4	28.6	0.2	13	38.2	0.7	32	32.0	1.7	63	28.1	3.3
Social status																								
1–2	41	0.8	77.4	4	1.2	7.5	2	0.9	3.8	-	-	-	-	-	-	1	2.7	1.9	-	-	-	5	2.2	9.4
3–4	418	7.7	73.1	43	12.7	7.5	35	16.5	6.1	22	11.5	3.8	-	-	-	2	5.4	0.3	19	19.0	3.3	33	14.3	5.8
5–6	2,138	39.5	83.6	117	34.5	4.6	89	42.0	3.5	65	34.0	2.5	6	46.2	0.2	20	54.1	0.8	39	39.0	1.5	82	35.7	3.2
7–8	2,315	42.8	84.9	133	39.2	4.9	69	32.5	2.5	83	43.5	3.0	7	53.8	0.3	9	24.3	0.3	30	30.0	1.1	81	35.2	3.0
9–10	497	9.2	79.8	42	12.4	6.7	17	8.0	2.7	21	11.0	3.4	-	-	-	5	13.5	0.8	12	12.0	1.9	29	12.6	4.7
Friends using cannabis																								
None	3,936	71.3	95.0	105	30.3	2.5	58	27.1	1.4	36	18.4	0.9	4	28.6	0.1	2	5.3	0.0	1	1.0	0.0	2	0.9	0.0
A few	1,471	26.7	72.8	189	54.5	9.4	134	62.3	6.6	109	55.6	5.4	4	28.6	0.1	17	44.7	0.8	31	30.4	1.5	65	27.7	3.2
About half	68	1.2	25.3	34	9.8	12.6	17	7.9	6.3	26	13.3	9.7	4	28.6	1.5	11	28.9	4.1	34	33.3	12.6	75	31.9	27.9
Many	33	0.6	19.1	17	4.9	9.8	1	0.5	0.6	22	11.2	12.7	2	14.3	1.2	4	10.5	2.3	26	25.5	15.0	68	28.9	39.3
(Almost) all	9	0.2	15.8	2	0.6	3.5	4	1.9	7.0	3	1.5	5.3	-	-	-	4	10.5	7.0	10	9.8	17.5	25	10.6	43.9
Alcohol use																								
No	4,075	73.4	93.1	155	44.4	3.5	50	23.3	1.1	41	20.9	0.9	7	50.0	0.2	11	28.9	0.3	17	16.8	0.4	21	8.9	0.5
Yes	1,477	26.6	63.5	194	55.6	8.3	165	76.7	7.1	155	79.1	6.7	7	50.0	0.3	27	71.1	1.2	84	83.2	3.6	216	91.1	9.3
Hookah use																								
No	5,521	99.3	88.3	242	69.5	3.9	185	86.4	3.0	106	53.8	1.7	11	78.6	0.2	17	44.7	0.3	81	79.4	1.3	88	37.8	1.4
Yes	37	0.7	8.2	106	30.5	23.4	29	13.6	6.4	91	46.2	20.1	3	21.4	0.7	21	55.3	4.6	21	50.6	4.6	145	62.2	32.0

Proportions of the reported variables within the different consumption groups (% col) and proportions of the consumption groups within the reported variables (% row) are displayed. Missings were excluded from proportions calculation: gender *n* = 134, age *n* = 15, migration background *n* = 242, subjective social status *n* = 180, friends using cannabis *n* = 48, current alcohol use *n* = 9, current hookah use *n* = 7. All reported variables differ significantly by consumption group with *p* <.001

### Associations between cannabis and combustible/e-cigarette use

Table 2 presents the proportion of current cannabis users among current combustible and/or e-cigarette users. Current use of e-cigarettes was associated with 42.8 (95%-CI, 23.5–82.6) greater odds to currently use cannabis compared to never combustible nor e-cigarette users, respectively for current use of combustible cigarettes with 185.9 (95%-CI, 108.0–347.7) and current dual use with 468.7 (95%-CI, 278.2–858.3) greater odds (aORs), showing an overall significant association between current combustible/e-cigarette and cannabis use.

**Table 2:** Proportions of current cannabis users among current combustible and/or e-cigarette users

Combustible/e-cigarette use	Never cannabis user		Current cannabis user		Total	
	N	%	N	%	N	%
Never combustible nor e-cigarette user	5,559	99.7	14	0.3	5,573	83.0
Current e-cigarette user	349	90.2	38	9.8	387	5.8
Current combustible cigarette user	215	67.8	102	32.2	317	4.7
Current dual user	197	45.4	237	54.6	434	6.5
Total	6,320	94.2	391	5.8		

### Risk perception of cannabis use

Assessment of differences in cannabis risk perception revealed a significant main effect of current combustible/e-cigarette use (F (3) = 160.9, *p* < .001). Regression coefficients and post-hoc estimates are reported in Table 3. The model explained 4.5% (adjusted R^2^) of variance. Among current combustible/e-cigarette users, dual users perceived the lowest risk, followed by combustible cigarette users and then e-cigarette users. Never combustible nor e-cigarette users perceived the highest risk of cannabis use. The difference between combustible cigarette users and dual users was not significant (*p* = .392).

**Table 3:** Linear model predicting perceived risk of cannabis use by current combustible/e-cigarette use (*N* = 6,307). The categorical predictor was dummy-coded with never users being the reference groups

Predictors	Cannabis use risk perception				Post-hoc estimates	
	β	95%-CI	t	p	M (SE)	95%-CI
Combustible/e-cigarette use						
Never combustible nor e-cigarette user (reference)					2.82 (0.01)	2.80, 2.84
Current e-cigarette user	−0.40	−0.51, −0.30	−7.48	<.001^***^	2.51 (0.04)	2.43, 2.59
Current combustible cigarette user	−0.70	−0.84, −0.57	−10.36	<.001^***^	2.28 (0.05)	2.18, 2.38
Current dual user	−0.86	−1.00, −0.72	−12.06	<.001^***^	2.16 (0.05)	2.06, 2.27

^***^ *p*.001

### Intention to use cannabis

Assessment of differences in intention to use cannabis revealed significant main effects of current combustible/e-cigarette use (F (3) = 261.5, *p* < .001) and cannabis risk perception (F (1) = 618.0, *p* < .001). Regression coefficients and post-hoc estimates are reported in Table 4. The model explained 22.5% (adjusted R^2^) of variance. Among never cannabis users, dual users of combustible and e-cigarettes reported the highest intention to use cannabis, followed by combustible cigarette users and e-cigarette users. Never combustible nor e-cigarette users reported the lowest intention to use cannabis. Perceived harm of cannabis correlated negatively with intention to use cannabis.

**Table 4:** Linear model predicting intention to use cannabis by current combustible/e-cigarette use and perceived risk of cannabis use among never cannabis users (*N* = 6,290). The categorical predictor was dummy-coded with never users being the reference groups

Predictors	Intention to use cannabis				Post-hoc estimates	
	β	95%-CI	t	p	M (SE)	95%-CI
Combustible/e-cigarette use						
Never combustible nor e-cigarette user (reference)					0.15 (0.00)	0.14, 0.16
Current e-cigarette user	0.59	0.49, 0.69	12.05	<.001^***^	0.37 (0.02)	0.34, 0.41
Current combustible cigarette user	0.96	0.84, 1.08	15.56	<.001^***^	0.51 (0.02)	0.47, 0.56
Current dual user	1.42	1.29, 1.54	21.85	<.001^***^	0.69 (0.02)	0.64, 0.74
Risk perception	−0.28	−0.30, −0.26	−24.86	<.001^***^		

^***^ *p*.001

### Sensitivity analyses

The results of complete-case regression models that are adjusted for control variables such as demographic variables, peers’ cannabis use, use of alcohol and hookah can be found in Table 5. After adjusting for control variables, the main effects shown in Tables 3 and 4 remained significant.

**Table 5:** Linear models predicting 1) perceived harm of cannabis use by current combustible/e-cigarette use (*N* = 5,820), and 2) intention to use cannabis by current combustible/e-cigarette use and perceived harm of cannabis use (*N* = 5,814). Categorical predictors were dummy-coded. Models are adjusted for demographic variables, alcohol use, hookah use, peers’ cannabis use and data source

Predictors	Cannabis use risk perception ^a^				Intention to use cannabis ^b^			
	β	95%-CI	t	p	β	95%-CI	t	p
Cigarette/e-cigarette use								
Never combustible nor e-cigarette user (reference)								
Current e-cigarette user	−0.20	−0.29, −0.11	−4.23	<.001^***^	0.32	0.21, 0.42	5.81	<.001^***^
Current combustible cigarette user	−0.33	−0.44, −0.23	−6.06	<.001^***^	0.70	0.58, 0.83	10.90	<.001^***^
Current dual user	−0.42	−0.54, −0.29	−6.59	<.001^***^	0.99	0.85, 1.14	13.32	<.001^***^
Risk perception	-	-	-	-	−0.24	−0.27, −0.22	−20.88	<.001^***^
Data source								
Methodenkoffer (reference)								
Quo Vadis	−0.06	−0.10, −0.02	−2.85	0.004^**^	0.11	0.06, 0.15	4.60	<.001^***^
Age	0.01	−0.01, 0.03	0.87	0.382	−0.02	−0.05, −0.00	−2.00	0.045^*^
Gender								
Female (reference)								
Male	−0.02	−0.06, 0.02	−0.95	0.343	−0.02	−0.06, 0.03	−0.79	0.429
Migration								
No (reference)								
Yes	0.09	0.05, 0.13	4.41	<.001^***^	0.03	−0.02, 0.08	1.07	0.287
Social status	0.03	0.02, 0.05	5.14	<.001^***^	0.01	−0.01, 0.04	1.12	0.264
Friends using cannabis	−0.14	−0.18, −0.11	−8.49	<.001^***^	0.17	0.15, 0.19	13.97	<.001^***^
Alcohol use								
Non-user (reference)								
Current alcohol user	−0.24	−0.28, −0.20	−9.43	<.001^***^	0.32	0.26, 0.37	12.01	<.001^***^
Hookah use								
Non-user (reference)								
Current hookah user	−0.00	−0.11, 0.11	−0.04	0.970	0.13	0.01, 0.26	2.05	0.040^*^

^a^adj. *R*^2^ = 8.3%

^b^adj. *R*^2^ = 27.6%

* *p*.05, ** *p*.01, *** *p*.001

## Discussion

In a large sample of young German adolescents, mainly 13- to 16-year-olds, 17.2% either used cannabis, combustible cigarettes, e-cigarettes, or any combination of the substances in a short-term period prior to the date of survey. Most prevalent with 12.2% within the past 30 days were e-cigarettes, followed by a 30-day prevalence of combustible cigarette use among 11.2%, and a prevalence of current cannabis use among 5.8% of the sample.

Current combustible/e-cigarette use was strongly associated with current cannabis use. Dual users of combustible and e-cigarettes had the highest probability to currently use cannabis with over half of them reporting current cannabis co-use, followed by current users of combustible cigarettes with one third reporting current cannabis use. Current users of e-cigarettes also had an increased probability to report current cannabis use compared to never combustible nor e-cigarette users, one in ten e-cigarette-only users currently used cannabis. Only a very small proportion of young adolescents (< 0.3% of total sample) reported current use of cannabis but never used combustible nor e-cigarettes.

Consistent with previous findings (Lopez-Quintero and Neumark 2010), we replicated that the perceived risk of cannabis use is negatively linked to the intention to use cannabis, supporting the motivational hypothesis (Grevenstein et al. 2015). Beyond previous findings, we showed that, among never cannabis users, the use of combustible and e-cigarette products is associated with cannabis-related variables: Adolescents that never used cannabis but were users of combustible or e-cigarettes perceived cannabis use as less harmful and were more likely to report a higher intention to use cannabis compared to never cannabis nor combustible/e-cigarette users. This association was strongest among dual users of combustible cigarettes and e-cigarettes, followed by combustible cigarette only users, and weakest among e-cigarette only users. This pattern is consistent with the patterns of co-use reported above as well as reported by previous studies (Strong et al. 2018; Kotz et al. 2024).

Our findings support the idea of perceiving cannabis use as a high-risk behaviour being a protective factor against the intention to use cannabis (Salloum et al. 2018; Grevenstein et al. 2015). This implicates that awareness campaigns addressing the harms of cannabis are a valid option to reduce cannabis use, especially as a primary prevention strategy among never users. Based on our results, the use of cigarettes and e-cigarettes should be considered in the design and addressing of cannabis prevention strategies. Young (e-)cigarette users seem to be a particularly vulnerable group for later cannabis use initiation and should be specifically addressed. Given these findings and taking into account the idea of common liabilities (Lemyre et al. 2019), it might make sense to combine preventive measures against cannabis with preventive measures against (e-)cigarettes. This might also involve a convergence of legislation and policy concerning tobacco and cannabis products. Although there have been efforts to restrict tobacco policies in Germany in recent years, e. g. ban on advertisement for combustible cigarettes (2022) and e-cigarettes (2024), combustible and e-cigarette products are for sale in every conventional kiosk or supermarket and in most cases cannot be overlooked in the checkout area. Contrary, cannabis was only partially legalized in April 2024 and its consumption, sale and possession are subject to strict regulations.

Our study shows relevant differences between combustible and e-cigarette users amongst all cannabis use variables (current use, risk perception, use intention). Considering the route of administration theory (Lemyre et al. 2019), one can assume that, among the co-users, combustible cigarettes are correlated with cannabis smoking and e-cigarettes with cannabis vaping. As shown by Kotz et al. (2024), in Germany smoking cannabis (92.4%) is much more prevalent compared to cannabis vaping (1.7%). They showed other administration routes being very rare, what seems to be consistent with the very small number of adolescents in our study who reported current cannabis but no current combustible or e-cigarette use (as mentioned above).

## Limitations

One limitation to our study is that we used cross-sectional data. Therefore, the causal interpretations of previously shown associations are limited. Further, the sample sizes between the (e-)cigarette groups differed widely. This is also why we did not adjust the models for covariates as missings would lead to further loss of data minimizing the number of observations within smaller groups. However, later sensitivity analyses revealed similar results. The number of control variables in our analyses is limited. Other variables, e. g. mental health, parenting styles (Lemyre et al. 2019), might influence the examined associations and should be considered in future research. These variables were not assessed in the survey and could therefore not be considered. There have been differences in measurement of intention to use cannabis between the two data sources with one survey examining intention to use cannabis next weekend and the next year while the other examined intention to use cannabis the next 2 weeks and the next half year. We accounted for this deviation by adjusting our models for the underlying data source. Another limitation is the measure of risk perception in generally without asking specifically for perceived risk on oneself. These two may diverge as users might be well informed about the possible risks without relating these risks to oneself. Our analyses focussed on combustible and e-cigarettes as these two are the most prevalent tobacco products among adolescents. Other tobacco products were either not assessed or, in the case of hookah, considered as control variable. Our study provides no information about the administration route of cannabis use. Further, we used self-reported data that can be biased (e. g. by social desirability) although the survey was anonymous.

## Conclusions

Among adolescents, the use of cannabis is strongly associated with the use of combustible cigarettes and e-cigarettes. This association not only can be examined for co-use of cannabis and combustible/e-cigarette products but also for perceived harm of cannabis use and intention to use cannabis in the immediate future. These findings show the need to develop prevention and intervention programs for young people addressing co-use of cannabis and combustible/e-cigarette products instead of solely targeting the use of cannabis. Further, although the main users of cannabis are often shown to be older adolescents and young adults, the present data shows that combustible cigarette, e-cigarette and cannabis use are already prevalent at younger adolescence and are highly correlated. Prevention programs should therefore be established at an early stage in school.

Further research should analyse causality between tobacco use, cannabis use, and perceived risk of cannabis use based on longitudinal studies and should consider a wider range of potential confounding variables (e. g. mental health, parenting style). Additionally, a closer look at perceived risk on oneself compared to perceived risk in general could be of interest within this context, as such results could have a substantial impact on development of prevention and intervention programs. Considering the changes in tobacco and cannabis policies since the data collection, it might be of interest to monitor changes in risk perception and consumption patterns over time.

## Supplementary Information

Supplementary Material 1.

## Data Availability

The datasets used and/or analysed during the current study are available from the corresponding author on reasonable request.
