# Supplementary material for: Association of cigarette and e-cigarette use with cannabis-related risk perceptions and intentions
Source: J Cannabis Res. 2025 May 31;7:31. doi: 10.1186/s42238-025-00288-6 (PMC12126890; doi:10.1186/s42238-025-00288-6)
Supplement: Supplementary file 1 — Supplementary Material 1. [file 42238_2025_288_MOESM1_ESM.docx]

**Appendix**

Appendix - Figure 1: Overview of sample acquisition and collection in the individual studies
